# Special Issue “Neurogenetics in Neurology”

**DOI:** 10.3390/ijms25021061

**Published:** 2024-01-15

**Authors:** Antonio Orlacchio

**Affiliations:** 1 Dipartimento di Medicina e Chirurgia, Università di Perugia, 06132 Perugia, Italy; a.orlacchio@hsantalucia.it; 2 Laboratorio di Neurogenetica, Centro Europeo di Ricerca sul Cervello (CERC), Istituto di Ricovero e Cura a Carattere Scientifico (IRCCS) Fondazione Santa Lucia, 00143 Rome, Italy

With the rapid developments in molecular genetics and genomics, this Special Issue collates works outlining ultra-modern scientific research.

The Special Issue entitled of the International Journal of Molecular Sciences includes six papers, among which is a research article, four reviews and a perspective piece, that highlight the remarkable advances made in the cognition of basic biological processes and their consequences for human health. Embracing disparate reasons, the six papers exhibited here cast a light on various aspects of molecular genetics and genomics, highlighting the tangled mutual influence of genetic information, cellular processes and physopathogenic mechanisms.

A key theme emerging from these works is the growing knowledge of the molecular mechanisms underlying various genetics-based diseases affecting the nervous system. Understanding of the pathogenesis of these diseases is increased by articulating strategies to use this knowledge to provide novel therapeutic tools. In their paper, Kuznitsov-Yanovsky et al. expose the slowed maturation of X-originating fragile neurons at the transcriptomic and functional levels because of delayed neuronal growth and maturation, identifying the related damaged neurodevelopmental pathways, i.e., mainly the TGF_/BMP signaling pathway. These results provide new insights into the molecular pathway by which fragile X mental retardation protein (FMRP) deficiency acts on the neuronal network development [1].

Moreover, the study by Monfrini et al., highlights that brain calcification can denote the epiphenomenon of many systemic and genetic conditions. An associated clinical appearance (such as dysmorphism, cutaneous abnormalities or immunological traits) can assist in the procedure of a differential diagnosis. Interestingly, several genes accountable for these forms are implicated in the mechanisms of angiogenesis and in the blood–brain barrier (BBB), suggesting common ways that could be blocked by therapeutic approaches to these conditions [2].

Improvements in technology, such as deep convolutional neural networks, have also enabled the comprehensive exploration of the molecular mechanisms underlying diverse diseases affecting the nervous system. The review by Dr. Cencioni et al., summarizes the existing information on glioblastoma multiforme (GBM) metabolism, with a detailed focus on the control of the Myc oncogene that, in turn, rules the activation of metabolic signals, establishing the formation of a GBM tumor [3,4,5].

Furthermore, Faravelli et al. exhibited the multifaced function of the survival motor neuron (SMN) protein and the influence of the loss of distinct roles of SMN in the pathogenesis of SMA. The weakening of snRNP assembly and the SMN complex has been strongly associated with the residual levels of SMN activity in cellular models of SMA and regulates downstream tissue-specific splicing alterations. The translational effects of these observations indicate that restoring the role of snRNP in animal models of SMA is sufficient to save the phenotype [6,7].

The biological and clinical importance of microRNAs (miRNAs or miRs) is clearly highlighted in the work of Kumar and Li., where they review the present advancement in miR-30c in the pathogenesis of various neurological disorders such as Alzheimer’s disease, Parkinson’s disease, multiple sclerosis and stroke, and its therapeutic capability. Bioinformatic analysis and experimental indication recommend that miR-30c controls many genes as targets that are linked with several pathogenic processes, such as autophagy, apoptosis, ER stress, inflammation, oxidative stress, thrombosis, and neurovascular function, thus contributing to the pathophysiology of diverse neurological diseases. In this context, the therapeutic targeting of miR-30c via synthetic mimics or inhibitors is under evaluation [8,9,10].

The last manuscript in this Special Issue by Mercurio et al. reviews recent papers producing long-range interaction maps (via HiC, RNApolII ChIA-PET, Capture-HiC or PLACseq) and overlapping the recognized long-range networking DNA segments with DNA sequence modifications associated with neurodevelopmental disorders (NDD) (such as schizophrenia, bipolar disorder, and autism) and traits (intelligence). Hence, long-range interaction maps, in combination with DNA changes noticed in association with NDD, can be used as “pointers” to detect novel candidate disease-applicable genes. The functional handling of the long-range communication network, including enhancers and promoters via CRISPR-Cas9-based approaches, is beginning to be probed for the functional meaning of recognized interactions and the enhancers and the genes involved, enlightening our understanding of neural growth and its pathology [11].

As indicated by these diverse contributions to the Special Issue, a common storyline appears: the complex connection between genetics, molecular mechanisms, and disease. These works cooperatively highlight the outstanding strides we have taken in solving the molecular complexities of diverse diseases, while also highlighting the prospects that stay ahead.

Overall, the papers in this Special Issue highlight the latest progress in molecular genetics and genomics and improve our existing knowledge of the key mechanisms in this field. These conclusions not only showcase the achievements of the past, but also pave the way for awakening possibilities of further investigation in the field of molecular genetics and genomics.

In closure, I would like to present my greatest thanks to the authors, reviewers, and the Editorial team for their precious contributions to this Special Issue. I hope that these manuscripts will not only improve our perception of molecular genetics and genomics, but also inspire further analysis and novelty in this dynamic and ever-evolving field.
